# Quality of life of cutaneous leishmaniasis suspected patients in the Ecuadorian Pacific and Amazon regions: a cross sectional study

**DOI:** 10.1186/s12879-022-07733-4

**Published:** 2022-09-24

**Authors:** Jacob Machiel Bezemer, Manuel Calvopiña Hinojosa, Andrea Estefania Corral Zabala, Fernando Ortega Pérez, Veronica Cristina Vargas Román, Henk Dirk Frederik Herman Schallig, Henry John Christiaan de Vries

**Affiliations:** 1 Fundación Misión Cristiana de Salud, Hospital Shell, Shell, Ecuador; 2 Laboratory for Experimental Parasitology, Department of Medical Microbiology and Infection Prevention, Amsterdam UMC Location University of Amsterdam, Amsterdam, The Netherlands; 3 Infectious Diseases Program, Amsterdam Institute for Infection and Immunity (AII), Amsterdam, The Netherlands; 4 Facultad de Ciencias de la Salud, Carrera de Medicina, OneHealth Research Group, Universidad de las Américas, Quito, Ecuador; 5 School of Public Health, College of Health Sciences, Universidad San Francisco de Quito, Quito, Ecuador; 6 Faculty of Social Sciences, Katholieke Universiteit Leuven, Leuven, Belgium; 7 Department of Dermatology, Amsterdam UMC Location University of Amsterdam, Amsterdam, The Netherlands; 8 Department of Infectious Diseases, Public Health Service Amsterdam, Center for Sexual Health, Amsterdam, the Netherlands

**Keywords:** Leishmaniasis, Cutaneous, Quality of life, Time-to-treatment, Geographic locations, Ecuador

## Abstract

**Background:**

Yearly, up to 1 million patients worldwide suffer from cutaneous leishmaniasis (CL). In Ecuador, CL affects an estimated 5000 patients annually. CL leads to reduced Health Related Quality of Life (HRQL) as a result of stigma in the Asian and Mediterranean contexts, but research is lacking for Ecuador. The objective of this study was to explore the influence of CL suspected lesions on the quality of life of patients in the Pacific and Amazon regions.

**Methods:**

Patients for this study were included in the Amazonian Napo, Pastaza, and Morona Santiago provinces and the Pacific region of the Pichincha province. Participating centers offered free of charge CL treatment. All patients suspected of CL and referred for a cutaneous smear slide microscopy examination were eligible. This study applied the Skindex-29 questionnaire, a generic tool to measure HRQL in patients with skin diseases. All statistical analysis was done with SPSS Statistics version 28.

**Results:**

The skindex-29 questionnaire was completed adequately by 279 patients who were included in this study. All patient groups from the Amazon scored significantly (P < 0.01) higher (indicating worse HRQL) on all the dimensions of the Skindex-29 questionnaire than Mestizo patients from the Pacific region. The percentage of patients with health seeking delay of less than a month was significantly (P < 0.01) lower in the Amazon region (38%) than in the Pacific (66%).

**Conclusions:**

The present study revealed that the influence of suspected CL lesions on the HRQL of patients in the Ecuadorian Amazon and Pacific depends on the geographic region more than on patient characteristics such as gender, age, number of lesions, lesion type, location of lesions, health seeking delay, or posterior confirmation of the *Leishmania* parasite. The health seeking delay in the Amazon might result from a lack of health infrastructure or related stigma. Together, the impaired HRQL and prolonged health seeking delay in the Amazon lead to prolonged suffering and a worse health outcome. Determinants of health seeking delay should be clarified in future studies and CL case finding must be improved. Moreover, HRQL analysis in other CL endemic regions could improve local health management.

**Supplementary Information:**

The online version contains supplementary material available at 10.1186/s12879-022-07733-4.

## Background

**Fig. 1:**
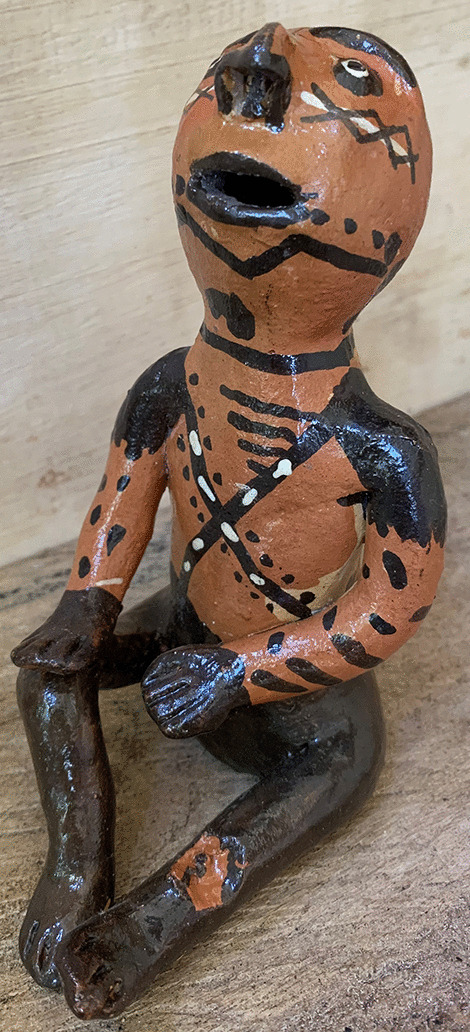
A traditional Ecuadorian Amazon Kichwa figurine from the Pastaza province depicts a patient with concomitant ML and CL

Yearly, up to 1 million patients worldwide are affected by cutaneous leishmaniasis (CL), and almost a third of those cases occur in South America [1, 2]. CL is a vector-borne parasitic disease, mainly characterized by cutaneous ulcers, and is considered a Neglected Tropical Disease by the World Health Organization (WHO) [2]. CL affects between 3900 and 6400 patients yearly in Ecuador, with clusters in the subtropical Pacific and Amazon regions. Mucosal Leishmaniasis (ML) occurs in approximately 2.5% of the Ecuadorian cases, mainly in the Amazon region [3, 4]. CL leads to reduced Health Related Quality of Life (HRQL) as a result of social and self-stigma, as has been established in the Asian and Mediterranean contexts [5]. In contrast, the few studies that assessed HRQL of CL patients in northern South America found no evidence for stigmatization [6, 7]. CL patients reported reduced self-esteem in the Ecuadorian subtropical Pacific in 1994 but no follow up was done [8]. Ecuador’s Amerindian population has been marginalized and discriminated against since colonial history, echoing in health access inequalities despite contemporary constitutional equality [9–11]. The Ecuadorian Amazon society is a mix of multiple cultures, and almost half of the population self-identifies as Amerindian, contrasting with the Pacific region, where more than 90% is Mestizo (of mixed Amerindian and European origin) [12, 13]. Amerindian patients are seldom included in HRQL studies of CL, although it is highly endemic in their Amazonian territory, and the presence of patients with destructive ML might affect the disease perception (see Fig. 1) [3, 14, 15]. Hence the need for HRQL studies that include Amerindian CL patients from the Amazon region.

## Methods

### Objectives

The main objective of this study was to explore the influence of CL suspected lesions on the quality of life of patients in the subtropical Pacific and Amazon regions with the hypothesis that there was self- and social stigmatization. As a secondary objective, we aimed to explore determinants of health-related quality of life.

### Participants

This study was part of a cross sectional project on CL suspected patients that included a quantitative questionnaire for HRQL, assessment of diagnostic tests, description of geographic *Leishmania* species distribution, and a multisite short-term ethnography.

Patients were included in private and public primary health care centers and hospitals in the Amazonian Napo, Pastaza, and Morona Santiago provinces and in the Pacific region of the Pichincha province. A part of the patients was included during community visits. All participating centers offered free of charge outpatient treatment for CL according to the guidelines of the Ecuadorian Ministry of Health (once daily intra-muscular meglumine antimoniate for 20 consecutive days) [16].

All CL suspected patients referred for a cutaneous smear slide microscopy examination in the participating centers were eligible to participate in the study. Participants were approached by doctors, nurses, or laboratory technicians during normal workflow before diagnostic sampling from CL suspected lesions. The patient or its legal representative filled out the HRQL questionnaire without knowledge of the test results. When needed, help from family members, a translator, or a health professional was allowed. The results were sent to a central data repository by mail service or delivered personally. Patients were included from January 2019 through June 2021. Patients who answered less than 75% of the questionnaire items were excluded from the study.

### Questionnaire and variables

The Skindex-29 questionnaire is a generic tool to measure HRQL in patients with skin diseases. It contains 29 questions related to three dimensions: 10 questions on the emotional dimension (e.g., I am worried, angry, or ashamed by my skin condition.), seven questions on symptoms (e.g., My skin condition hurts, irritates, or burns.), and 12 questions on functioning (e.g., My skin condition affects my social life). Following a 5-point Likert scale for each question, patients can respond either: Never, rarely, sometimes, often, or all the time [17]. The Skindex-29 questionnaire has been translated and validated in more than ten languages in North and South American, European, Asian, and African cultures [18–23]. This study applied the Spanish version, which was previously validated in Colombia [24].

In addition, the following variables were recorded: Gender (male or female), age in years, ethnicity (as recognized by the Ecuadorian government [25]), perceived place of infection, number of lesions separated by healthy skin, type of lesion(s) (ulcer, nodular, or other), lesion location (indicated on a person image), health seeking delay (in weeks, months, or years), and the result of the smear slide microscopy and Polymerase Chain Reaction (PCR) (positive or negative).

### Analysis

The Skindex-29 questionnaire results and the other variables were entered in the data management platform Castor EDC (https://data.castoredc.com). Data entry was done in duplicate by JB and AC and validated with calculation fields. The categorical responses were transformed into linear variables on a scale from 0 to 100, with 0 indicating no impairment and 100 indicating the worst HRQL, as described elsewhere [17]. Averages were calculated per dimension and for the total. Missing Skindex-29 answers were replaced by the average score of the corresponding dimension. If a second variable was missing, the patient was subsequently excluded from that specific comparison.

Confirmed (positive for either microscopy, PCR, or both) versus non-confirmed CL patients were compared to assess the feasibility of generalizing Skindex-29 scores for the entire patient group. Pacific and Amazonian regions are divided by the Ecuadorian highlands, where leishmaniasis is rare [26]. The prevalent *Leishmania* species, vector-human interaction, and social structure in the two regions differ and were therefore analyzed separately and compared [4, 26, 27]. To allow comparisons, patients were divided into four linguistic groups: Spanish speaking Mestizos, Kichwa (Amazon Kichwa, Andwa, and Zapara), Chicham (Shuar, Achuar, and Shiwiar), and other (Waorani, white, and Afro-Ecuadorian). Patients with multiple lesion types (e.g. nodules and ulcers) were categorized as ulcer type if at least one ulcer was present because then we anticipated worse HRQL [28]. The body location of lesions was categorized as: ‘head and face’, ‘upper limbs’, ‘lower limbs’, or ‘trunk’ as in a former study in Surinam that found an association between body location of the lesions with Skindex-29 scores in CL patients [14].

The sample size was not calculated but based on convenience sampling. All statistical analysis was done with SPSS Statistics version 28 [29]. Mean Skindex-29 scores were assessed for statistical significance with the independent samples T test or Oneway Anova. Other variables were compared with the independent samples Proportions (Wald) or T test. Statistical significance was defined as (two-sided) P value < 0.05.

## Results

### Participants

A total of 324 patients provided written informed consent. Four patients presented exclusively with mucosal lesions and were therefore excluded. Forty-one patients filled in less than 22 items of the questionnaire and were excluded. The skindex-29 questionnaire was completed adequately by 279 patients who were included in this study. Less than 2% of the responses were missing in the remaining questionnaires.

### Baseline characteristics

The majority of the patients (58%) were male, and the mean age was 28 years, ranging from 0 to 88 years. 153 (55%) patients were included from the subtropical Pacific region and 126 (45%) from the Amazon region. 192 (69%) patients were Mestizos, and 205 (74%) had one lesion. Most patients (91%) presented with at least one ulcerative lesion and had lesions on the upper or lower limbs. Health seeking delay was less than a month in 149 (54%) patients (Additional file 1: Table S1).

### Confirmed CL cases

*Leishmania* parasites were identified with microscopy and/or PCR in the skin lesions of 208 (75%) patients. Of the confirmed cases 84 (40%) presented with lesions on the upper limbs and 56 (27%) on the lower limbs compared to 15 (21%) and 30 (43%) respectively of the non-confirmed cases. This difference was statistically significant (P < 0.01 for upper limbs and P = 0.02 for lower limbs). Confirmed cases did not differ significantly in gender, age, ethnicity, region of infection, number of lesions, lesion type, and health seeking delay from non-confirmed cases (Table 1). Confirmed cases scored lower on the Skindex-29 questionnaire in both the Pacific and Amazon regions, but the difference was not statistically significant (Table 2).

**Table 1:** Characteristics of the study population (N = 279) of cutaneous leishmaniasis confirmed and non-confirmed patients in the Ecuadorian subtropical Pacific and Amazon regions from January 2019 through June 2021

Characteristic (N missing for variable)	CL confirmed^a^	CL non-confirmed	Two-sided P^b^	All patients
Number (%)	208 (75)	71 (25)		279 (100)
Male gender (%)	118 (57)	43 (61)	0.57	161 (58)
Age in years (1)				
Mean ± SD	27.3 ± 19.4	30.6 ± 21.4	0.23	28.1 ± 19.9
Range	0.1—88	1.0—75		0.1–88.0
Ethnicity^c^ (0)				
Pacific Mestizo (%)	121 (100)^d^	32 (100)^d^		153 (100)
Amazon Mestizo (%)	23 (26)	16 (41)	0.11	39 (31)
Amazon Kichwa (%)	26 (28)	11 (30)	0.85	37 (29)
Amazon Chicham (%)	34 (39)	10 (26)	0.12	44 (44)
Amazon Other (%)	4 (5)	2 (5)	0.90	6 (5)
Geographic region (0)				
Pacific (%)	121 (58)	32 (45)	0.06	153 (55)
Amazon (%)	87 (42)	39 (55)	0.06	126 (45)
Number of lesions (1)				
1 (%)	151 (73)	54 (77)	0.44	205 (74)
2 (%)	37 (18)	8 (11)	0.17	45 (16)
≥ 3 (%)	20 (10)	8 (11)	0.67	28 (10)
Lesion type (1)				
Ulcer (%)	135 (89)	117 (93)	0.25	252 (91)
Location of the lesions (1)				
Head and face (%)	41 (20)	12 (17)	0.63	53 (19)
Upper limbs (%)	84 (40)	15 (21)	< 0.01^e^	99 (36)
Lower limbs (%)	56 (27)	30 (43)	0.02^e^	86 (31)
Trunk (%)	27 (13)	13 (19)	0.28	40 (14)
Health seeking delay (2)				
1–4 weeks (%)	114 (55)	35 (50)	0.46	149 (54)
1–2 months (%)	42 (20)	12 (17)	0.55	54 (19)
≥ 2 months (%)	51 (25)	23 (33)	0.20	74 (27)

^a^Either by microscopy, PCR or both

^b^Comparing CL confirmed and non-confirmed cases with the independent samples proportions (Wald) or T test

^c^Kichwa (Amazon Kichwa, Andwa, and Zapara) and Chicham (Shuar, Achuar, and Shiwiar) are linguistic groups

^d^All patients from the Pacific region were Mestizos

^e^Statistically significant difference

**Table 2:** Mean Skindex-29 scores of patients (N = 279) suspected of having cutaneous leishmaniasis in the Ecuadorian subtropical Pacific and Amazon regions from January 2019 through June 2021

Skindex-29 dimension:	Emotions (SE)	Symptoms (SE)	Functioning (SE)	Total (SE)
Pacific				
Confirmed leishmaniasis (n = 121)	31.7 (2.0)	37.9 (2.3)	17.4 (2.0)	27.3 (1.8)
Non-confirmed leishmaniasis (n = 32)	33.0 (4.3)	41.2 (4.9)	19.1 (4.5)	29.2 (4.2)
Two-sided P^a^	0.78	0.52	0.71	0.64
Amazon				
Confirmed leishmaniasis (n = 87)	50.8 (2.5)	53.3 (2.3)	41.8 (2.8)	47.7 (2.3)
Non-confirmed leishmaniasis (n = 39)	58.5 (4.1)	61.9 (4.1)	50.7 (4.7)	56.1 (4.0)
Two-sided P^a^	0.10	0.06	0.09	0.06

*SE* standard error

^a^Comparing mean Skindex-29 scores of confirmed and non-confirmed cases with the independent samples T test

### HRQL of CL suspected patients in the Pacific and Amazon regions

The percentage of males (52%) included in the Pacific region was significantly lower than in the Amazon (64%). The percentage of patients with age 0–12 (31%) was significantly higher in the Pacific region than in the Amazon (19%) and the percentage from the age group ≥ 40 was significantly lower (18 and 33% respectively). In the Pacific region, 100% of the patients were Mestizo but in the Amazon, the majority of patients were either from the Kichwa (29%) or Chicham (35%) linguistic groups. The percentage of patients with lesions on the head or face was significantly less in the Amazon region (13%) than in the Pacific (24%). The percentage with health seeking delay of less than a month was significantly (P < 0.01 on the independent proportions test) lower in the Amazon region (38%) than in the Pacific (66%), but significantly (P < 0.01) higher than in the ≥ 2 months delay group (resp. 39% vs 16%). Patients from the Pacific and Amazon regions presented no significant differences in lesion type or number of lesions (Table 3).

**Table 3:** Characteristics of the study population (N = 279) of suspected cutaneous leishmaniasis patients in the Ecuadorian subtropical Pacific and Amazon regions from January 2019 through June 2021

Region of contagion:	Pacific	Amazon	Two-sided P^a^
Characteristics (N missing for variable)			
N (%)	153 (55)	126 (45)	
Males (%)	80 (52)	81 (64)	0.04^b^
Age quartiles (1)			
0–12 (%)	47 (31)	24 (19)	0.02^b^
13–22 (%)	37 (24)	30 (24)	0.92
23–39 (%)	40 (26)	31 (25)	0.74
40–88 (%)	28 (18)	41 (33)	< 0.01^b^
Ethnicity^c^ (0)			
Mestizo (%)	153 (100)	39 (31)	0.00^b^
Kichwa (%)	0 (0)	37 (29)	< 0.01^b^
Chicham (%)	0 (0)	44 (35)	< 0.01^b^
Other (%)	0 (0)	6 (5)	0.01^b^
Clinical presentation (1)			
Mean number of lesions (range)	1.5 (1–8)	1.6 (1–10)	0.36
Lesion type: ulcer (%)	135 (89)	117 (93)	0.24
Location of lesions (1)			
Head and face (%)	37 (24)	16 (13)	0.01^b^
Upper limbs (%)	55 (36)	44 (35)	0.83
Lower limbs (%)	42 (28)	44 (35)	0.19
Trunk (%)	18 (12)	22 (18)	0.18
Health seeking delay (2)			
1–4 weeks (%)	101 (66)	48 (38)	< 0.01^b^
1–2 months (%)	26 (17)	28 (22)	0.27
≥ 2 months (%)	25 (16)	49 (39)	< 0.01^b^

^a^Comparing Pacific and Amazonian patients with the independent samples proportions (Wald) or T test

^b^Statistically significant difference

^c^Kichwa (Amazon Kichwa, Andwa, and Zapara) and Chicham (Shuar, Achuar, and Shiwiar) are linguistic groups

Mean Skindex-29 scores were not significantly different between males and females and, except for the functioning dimension, between age quartiles. All patient groups (Amerindian and Mestizo) from the Amazon scored significantly higher on all the dimensions of the Skindex-29 questionnaire than Mestizo patients from the Pacific region. Amazon Amerindian patient groups scored higher than Mestizos on all the dimensions, but the differences were not significant in the Post-Hoc tests. The mean difference between mean Skindex-29 scores in the Pacific and Amazon regions was highest on the functioning dimension, although not statistically significant. Mean Skindex-29 scores were not significantly different between patients with one lesion or more than one lesion and, except for the total score, between those with or without ulcers. The location of the lesion had no significant influence on the total patients’ mean Skindex-29 score nor on the emotions or symptoms dimensions. Body location was significantly (P = 0.05 on Oneway ANOVA) associated with the mean functioning Skindex-29 score, although not significant on the Post-Hoc tests. Patients with health seeking delay of less than a month, scored significantly lower on the emotions and functioning dimensions but not on the symptoms dimension. Mean Skindex-29 scores are shown in Table 4.

**Table 4:** Mean Skindex-29 scores of the study population (N = 279) of patients with suspected localized cutaneous leishmaniasis in the Ecuadorian subtropical Pacific and Amazon regions from January 2019 through June 2021

Skindex-29 dimension: characteristic (N missing for variable)	Emotions (SE)	Symptoms (SE)	Functioning (SE)	Total (SE)
Gender (0)				
Male (n = 161)	43.1 (2.1)	46.9 (2.0)	32.5 (2.3)	39.6 (2.0)
Female (n = 118)	39.5 (2.2)	45.9 (2.4)	26.4 (2.4)	35.6 (2.1)
P-value^a^	0.25	0.77	0.08	0.18
Age quartiles (1)				
0–12 (n = 71)^b^	40.2 (3.2)	45.7 (3.4)	24.5 (3.1)	35.0 (2.9)
13–22 (n = 67)	39.5 (2.9)	42.4 (2.7)	25.9 (3.0)	34.6 (2.5)
23–39 (n = 71)	41.2 (3.3)	45.4 (3.2)	30.9 (3.7)	37.9 (3.2)
40–88 (n = 71)	44.7 (2.8)	51.8 (2.9)	37.5 (3.4)^c^	43.5 (2.8)
P-value^a^	0.63	0.19	0.03^d^	0.12
Pacific vs Amazon^e^ (0)				
Pacific Mestizo (n = 153)^b^	32.0 (1.8)	38.6 (2.1)	17.8 (1.8)	27.7 (1.7)
Amazon Mestizo (n = 39)	46.4 (4.0)^c^	52.8 (3.8)^c^	34.0 (4.4)^c^	42.8 (3.8)^c^
Amazon Chicham (n = 44)	55.0 (3.3)^c^	57.6 (3.7)^c^	47.8 (3.7)^c^	52.7 (3.2)^c^
Amazon Kichwa (n = 37)	58.3 (3.9)^c^	58.2 (3.6)^c^	50.8 (4.3)^c^	55.2 (3.5)^c^
Amazon Other (n = 6)	52.9 (13.7)	50.6 (9.6)^c^	51.0 (15.9)	51.6 (13.2)
P-value^a^	< 0.01^d^	< 0.01^d^	< 0.01^d^	< 0.01^d^
Mean difference Pacific vs Amazon	21.3 (2.8)	17.4 (2.9)	26.8 (3.0)	22.6 (2.6)
95% Confidence Interval^a^ of difference	15.7—26.8	11.6—23.2	20.8—32.8	17.5—27.7
Number of lesions (0)				
1 lesion (n = 205)	40.5 (1.8)	45.5 (1.8)	29.0 (2.0)	37.0 (1.7)
> 1 lesion (n = 74)	44.4 (2.9)	49.1 (3.0)	32.2 (3.2)	40.5 (2.8)
P-value^a^	0.27	0.31	0.41	0.29
Lesion type (1)				
Ulcer (n = 252)	42.2 (1.6)	47.3 (1.6)	30.7 (1.7)	38.7 (1.5)
Non-ulcer (n = 26)	33.5 (6.0)	37.1 (5.7)	20.3 (6.0)	28.9 (5.6)
P-value^a^	0.10	0.06	0.07	0.05^d^
Location of the lesion (1)				
Head and face (n = 53)	41.2 (3.8)	45.8 (4.0)	28.3 (4.2)	37.0 (3.7)
Upper limbs (n = 99)	40.6 (2.5)	42.2 (2.5)	25.9 (2.5)	34.9 (2.2)
Lower Limbs (n = 86)	41.4 (2.7)	48.7 (2.6)	30.7 (3.0)	38.7 (2.6)
Trunk (n = 40)	43.6 (3.9)	52.1 (4.4)	38.9 (4.9)	43.7 (4.0)
P-value^a^	0.57	0.11	0.05^d^	0.12
Health seeking delay (2)				
1–4 weeks (n = 149)^b^	37.4 (2.1)	43.6 (2.1)	25.5 (2.2)	34.0 (1.9)
1–2 months (n = 54)	46.9 (3.2)^c^	50.1 (3.7)	32.4 (3.6)	41.7 (3.2)
≥ 2 months (n = 74)	45.7 (2.9)	49.5 (3.0)	36.5 (3.5)^c^	42.8 (2.9)^c^
P-value^a^	0.02^d^	0.14	0.02^d^	0.02^d^

*SE* Standard Error

^a^With Oneway ANOVA or independent samples T test

^b^Reference category

^c^Statistically significant difference on the Post-Hoc test compared to the reference category

^d^Statistically significant

^e^Kichwa (Amazon Kichwa, Andwa, and Zapara) and Chicham (Shuar, Achuar, and Shiwiar) are linguistic groups

## Discussion

The present study revealed that the influence of suspected CL lesions on the HRQL of patients in the Ecuadorian subtropical Pacific and Amazon depends on the geographic region of infection more than on patient characteristics such as gender, age, number of lesions, lesion type, location of lesions, health seeking delay, or posterior confirmation of the *Leishmania* parasite. Moreover, HRQL was worse on all three dimensions of the Skindex-29 questionnaire in the Mestizo, Kichwa, and Chicham patient groups from the Amazon region compared to the subtropical Pacific.

Younger age (0–12 years), location of lesions (category not specified), and short health seeking delay (1–4 weeks) were also associated with lower scores on the functioning dimension of the Skindex-29 questionnaire. This could be explained by confounding as the patients from the Amazon region differed significantly from Pacific patients in these categories, and the mean difference in Skindex-29 scores between the regions was the highest on the functioning dimension. The proportion of patients in the 1–4 weeks health seeking delay category was almost double in the Pacific group, with better HRQL on the emotions dimension, compared to the Amazon group. Therefore, the association of the 1–2 months health seeking delay category with lower HRQL on the emotions dimension could be explained by confounding.

The association of ulcerative lesions with higher total Skindex-29 scores could be explained by the additional influence of the interruption of skin continuity in ulcerative skin diseases, as seen in another study involving leg ulcers [28]. Nevertheless, the difference in mean Skindex-29 scores between patients with ulcerative and non-ulcerative lesions was not significant on the separate dimensions.

The worse HRQL of patients from the Amazon region might result from confounding variables that were not included in our data. These unknown variables could be elucidated through qualitative research. We performed structured interviews with 30 of our participants, and the data will be reported elsewhere [30]. Stigma expressions from the qualitative interviews seem to be the best explanation for the significantly worse HRQL in our CL suspected patients from the Amazon. Our results suggest that the expressions of social and self-stigma might have a significant and widespread influence on HRQL in all its dimensions.

A Canadian study of 51 hidradenitis suppurativa patients showed that the Skindex-29 questionnaire can detect stigma expressions evoked by non-visual disease characteristics. Patients with malodor scored significantly higher on all the dimensions of the Skindex-29 questionnaire, although there was no significant difference in the Dermatology Life Quality Index [23]. Malodor might be one of the causes of the impaired HRQL in our patient group.

Health seeking delay and subsequent time to treatment of Amazon patients were significantly longer. The health seeking delay might result from transportation difficulties from remote jungle communities, lack of recourses, discrimination, failing diagnostic tests, and/or stigmatization as occurs with leprosy patients [31]. During the qualitative interviews, patients with shorter health seeking delays indicated worse stigma expressions [30]. Therefore, our study might even underestimate the HRQL impairment in Amazonian patients.

The Amazon patient group had significantly fewer lesions on the head and face than in the Pacific region, probably because of different biting patterns of the *Lutzomyia* vector in older humans [26, 32]. Additionally, the percentage of males (probably going shirtless more often) in the Amazon was significantly higher than in the Pacific, a finding that is in agreement with other studies and should be explained by the predominance of hunters and farmers in the Amazon CL patient group contrasted to domestic transmission in the Pacific [4, 33, 34].

Our study has some limitations: The Skindex-29 questionnaire has not been validated for use by parents for their children, but our data show no significantly different scores between the 0–12, 13–22, and 23–39 age groups, suggesting that it was valid to include all patients in the analysis [17, 24]. Second, this study used the Spanish version of the Skindex-29 questionnaire as validated in Colombia [24]. Nevertheless, many of the included Amerindian patients were non-native Spanish speakers and questions were translated by the health professional or a translator. This might have influenced the Skindex-29 scores of Kichwa and Chicham patients. We recommend the validation of quantitative HRQL questionnaires such as the Skindex-29 in Amerindian populations. Lastly, additional information on the ulcers (e.g. ulcer smell, presence of liquid discharge, and diameter) would have been of value.

We consider that the results of this study might be fairly generalizable for the patient populations in the Ecuadorian Amazon and Pacific areas because patients were included both from the public and private health care system, including representative Amerindian groups. Therefore, health authorities should strengthen their efforts to improve CL case detection in the Amazon and secure prompt treatment initiation. Additionally, the causes of health seeking delay should be clarified in future studies of health seeking behaviors combining quantitative and qualitative methods.

The Kichwa and Chicham linguistic groups extend into the Peruvian Amazon, where similar HRQL impairment in CL patients might occur as in Ecuador [13]. On the other hand, patients with suspected CL lesions in other regions could also have different HRQL outcomes. Hence, we recommend that research including quantitative questionnaires, such as the Skindex-29, combined with qualitative interviews should be considered for CL endemic countries.

## Conclusion

Suspected CL patients from the Ecuadorian Amazon report significantly impaired HRQL compared to their counterparts in the Pacific region. Additionally, Amazonian patients have significant health seeking delay, leading to prolonged suffering and a worse health outcome. Determinants of health seeking delay should be clarified in future studies, including quantitative and qualitative methods, and CL case detection and management by health authorities must be improved. Moreover, HRQL analysis in other CL endemic regions could improve local health management.

## Supplementary Information

**Additional file 1: Table S1.** Skindex-29 results. Individual patient variables and Skindex-29 scores.

## Data Availability

Al data generated or analyzed during this study are included in this published article and its supplementary information files.

## Abbreviations

CL: Cutaneous leishmaniasis
HRQL: Health Related Quality of Life
ML: Mucosal leishmaniasis
